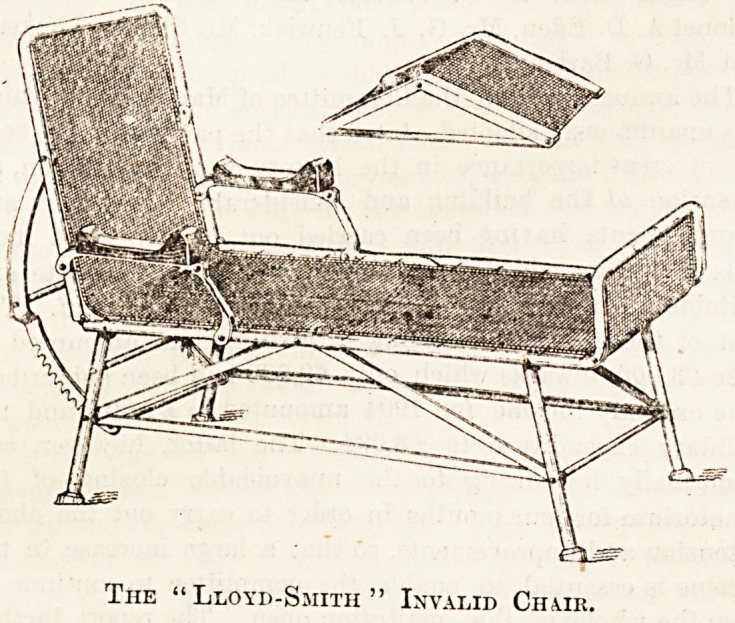# Practical Departments

**Published:** 1905-05-27

**Authors:** 


### PRACTICAL DEPARTMENTS.

### THE "LLOYD-SMITH" SANATORIUM CHAIR.

This is a chair which is especially suitable for sanatorium use, as it is a combination of a convenient carrying chair and comfortable couch, so that a patient when unable to walk can be removed from bed within doors to couch out of-doors without exertion. It has been adapted from one of similar design in use at Berlitz, Berlin, by the medical superintendent of the New Crossley Sanatorium, Delamere Forest. The illustration shows the general construction of the chair, the frame of which is made in wrought and malleable iron, with cast-iron feet, and polished wooden arm rests, and wire-wove web for the patient to rest upon. The back support is adjustable to any angle. The knee-rest is also movable in the same way. The chair can be conveniently used for carrying purposes, and is suitable of course for the use of all invalids. It is made by Messrs. Chorlton and Co., of Manchester, who are also makers of all kinds of bedsteads, amongst these being

THE "LLOYD-SMITH" INVALID CHAIR.

one also supplied to the Crossley Sanatorium and called the "Delamere." This bedstead is made of welded tubing with riveted rails, and is of great strength and rigidity. It is fitted with a strong galvanised diamond spring mesh, and has rubber-tyred castors. The construction is simple and there are no crevices for dust and it would be difficult to secure a bed of equal strength with fewer projections.

## Figures and Tables

**Figure f1:**